# Uropathogens’ AMR in Male and Female Romanian Population—A Bi-Center Analysis over 1 Year

**DOI:** 10.3390/medicina62050866

**Published:** 2026-04-30

**Authors:** Răzvan-Ionuț Popescu, Cătălin Nechita, Răzvan-Cosmin Petca, Cristian Mareș, Aida Petca, Cătălin Babiță, Viorel Jinga

**Affiliations:** 1 Department of Urology, ”Carol Davila” University of Medicine and Pharmacy, 8 Eroii Sanitari Blvd., 050474 Bucharest, Romania; dr.razvanp@gmail.com (R.-I.P.); ovidiu-catalin.nechita@drd.umfcd.ro (C.N.); dr.marescristian@gmail.com (C.M.); catalinbabita@gmail.com (C.B.); vioreljinga@yahoo.com (V.J.); 2 Department of Urology, “Prof. Dr. Th. Burghele” Clinical Hospital, 20 Panduri Str., 050659 Bucharest, Romania; 3 Department of Urology, “Saint John” Clinical Emergency Hospital, 13 Vitan-Barzesti Str., 042122 Bucharest, Romania; 4 Department of Obstetrics and Gynecology, ”Carol Davila” University of Medicine and Pharmacy, 8 Eroii Sanitari Blvd., 050474 Bucharest, Romania; 5 Department of Obstetrics and Gynecology, CF2 Clinical Hospital, 63 Marasti Blvd., 011464 Bucharest, Romania

**Keywords:** urinary tract infections, AMR, *E. coli*, *Klebsiella*, *Enterococcus*, male

## Abstract

*Background and Objectives:* Urinary Tract Infections (UTIs) are a major concern worldwide due to increasing antimicrobial resistance. Even though sex-based differences in antimicrobial resistance are recognized worldwide, there is a lack of data in the literature. This study aims to evaluate differences in the distribution of uropathogens and antibiotic resistance across large groups of Romanian males and females. *Materials and Methods:* This retrospective descriptive analysis included 2567 positive urine cultures collected over a 1-year period from hospitalized and outpatient patients at two representative urology centers in Bucharest. Only urine tests with ≥10^5^ CFU/mL and monomicrobial growth were included. *Results:* The sex distribution showed a predominance of male patients (62.2%). Also, their age tended to be higher than that of females. *Escherichia coli* remained the most common pathogen, with a higher prevalence in females (54.08% vs. 32.54%), while *Klebsiella* and *Pseudomonas* were more frequently noted in males. The Gram-negative analysis revealed higher resistance rates in male patients, particularly for common antibiotics such as amoxicillin-clavulanic acid (50.37% vs. 35.77%), trimethoprim-sulfamethoxazole (46.69% vs. 34.64%), and levofloxacin (45.55% vs. 34.71%). Notably, carbapenem resistance in *Klebsiella* exceeded 30% in males, indicating major concerns about multidrug resistance in this case. In contrast, Gram-positive bacteria showed more stable resistance patterns among the studied groups. Preserved sensitivity was found to linezolid, vancomycin, fosfomycin, and nitrofurantoin. *Conclusion:* These findings demonstrate clinically relevant sex-based differences in both pathogen distribution and antimicrobial resistance, particularly among Gram-negative uropathogens. Also, it highlights the importance of developing sex-adapted antibiotic strategies in conjunction with the local epidemiological data.

## 1. Introduction

Globally, data indicate that urinary tract infections (UTIs) are among the most common bacterial infections, both in the community and in healthcare settings [1,2]. Data from the 2019 Global Burden of Disease Study highlight the significant burden of urinary tract infections worldwide, with an estimated 404.61 million incident cases and 236,790 associated deaths. In addition, the global burden of disease, expressed in disability-adjusted life years (DALYs), was approximately 520,200 [3]. According to recent analyses using the GBD 2021 database, the number of UTI cases has increased significantly between 1990 and 2021. Thus, the age-standardized incidence rate is 5531.88 per 100,000 inhabitants, and the total number of cases reaches 4.49 billion [4]. These data support the fact that UTIs are a public health problem and an increasing clinical burden, and remain an important factor in prescribing antibiotics in multiple medical specialties [5].

Beyond the epidemiological impact, UTIs also generate a significant socioeconomic impact. They are the most common outpatient infections, affecting approximately 50–60% of adult women during their lifetime [6]. The prevalence of UTIs increases progressively with age, almost doubling in women aged 65 and older [7]. In the hospital setting, these infections are associated with higher mortality and significant healthcare costs. At the same time, recurrent episodes significantly affect quality of life and place a constant strain on the healthcare system [8].

Urinary tract infections have well-documented gender differences in clinical presentation, epidemiology, and pathophysiology [9]. Women are more vulnerable, particularly because of the anatomical proximity of the rectum to the urethral meatus. The female urethra is shorter, which favors bacterial colonization. In this context, *Escherichia coli* is responsible for approximately 75–95% of uncomplicated UTIs [1].

Risk factors such as anatomical features of the urinary tract and postmenopausal estrogen deficiency contribute significantly to the increased susceptibility of women to UTIs. In contrast, the incidence of UTIs in men is considerably reduced by the greater length of the male urethra and the protective effect of prostatic secretions with antibacterial properties. When UTIs occur in men, they are generally classified as complicated infections [1,9].

The increase in the incidence of UTIs in men with age is largely attributed to prostatic pathology. Benign prostatic hyperplasia (BPH) has a histological prevalence estimated at 50–60% among men aged 60 years, rising to over 80% in those aged 70 years or older [10]. The subvesical obstruction associated with BPH favours urinary stasis and creates conditions conducive to bacterial colonization. Thus, BPH contributes to the occurrence of complicated and recurrent urinary tract infections [10]. In this context, repeated urinary tract instrumentation procedures, the use of bladder catheters, and repeated exposure to antibiotic therapy among these patients may contribute to influencing local uropathogens, favoring the emergence and selection of strains with increased antibiotic resistance [11].

The relevance of these differences extends beyond epidemiological implications to their influence on the distribution of pathogens and antimicrobial resistance profiles [11]. Comparative studies between male and female patients in terms of uropathogens have highlighted a lower overall sensitivity to antibiotics in the case of male isolates. The most significant discrepancies are observed between *E. coli* and *Klebsiella* pneumoniae, especially regarding ceftazidime, aztreonam, trimethoprim-sulfamethoxazole, and fluoroquinolones [12].

To improve assessment of the uropathogens involved and guide selection of empirical antibiotic therapy, a gender- and age-stratified approach is useful [9].

Although these differences are well documented, recommendations for empirical therapy of urinary tract infections have predominantly been based on studies in female populations [13]. Current guidelines suggest that patients of both sexes should be treated with the same antimicrobial class, with the only difference being the duration of treatment, without significant adjustment based on gender [14]. This is a significant limitation in the context of increasing antibiotic resistance, where the inclusion of gender-stratified data could influence the choice of empirical antibiotic therapy [15].

Antimicrobial resistance (AMR) has increased considerably in recent decades among uropathogens [16,17]. According to European surveillance data from EARS-Net, high rates of antibiotic resistance persist in EU/EEA countries. An approximately 50% increase in carbapenem resistance among *Klebsiella* pneumoniae was observed between 2019 and 2022 [16]. Eastern European countries, including Romania, consistently rank among the regions with the highest rates of antimicrobial resistance on the continent. Resistance of Gram-negative uropathogens to fluoroquinolones and third-generation cephalosporins remains a major public health concern. Recent data from a study in Romania indicate that *E. coli* has developed progressively increased antibiotic resistance to amoxicillin-clavulanic acid and trimethoprim-sulfamethoxazole, as well as worrying rates of carbapenem resistance in *Klebsiella pneumoniae* [17].

Given this context, which represents an important public health issue, we performed a stratified analysis by sex of patients regarding the distribution of uropathogens and antimicrobial resistance profiles. Data were collected over a year from two tertiary urology centers in Romania. The objective of the study is to identify and characterize clinically relevant differences in pathogen prevalence and resistance patterns, to establish gender-adapted empirical antibiotic therapy and to support antimicrobial management strategies at local and regional levels.

## 2. Materials and Methods

This is an observational, retrospective, descriptive study. Positive urine samples collected from male and female patients at two tertiary urology centers in Bucharest, Romania: “Prof. Dr. Th. Burghele” Clinical Hospital and “Sfantul Ioan” Clinical Emergency Hospital, were analyzed and compared accordingly. Both hospitals serve as reference centers for urologic treatment and provide management for a wide range of pathologies, including stone disease, cancers, functional urology, and other benign conditions.

Between 1 January 2024 and 31 December 2024, all the positive urine tests were collected and analyzed. The study included both hospitalized cases and outpatients, and the samples were processed in the microbiological laboratories of each center, with the same antibiotic testing used to determine resistance and sensitivity.

According to the Ethical Committee policies, all patients received a similar informed general and focused consent form used by all the academic hospitals in Romania. As both centers have been involved in reporting data on bacterial prevalence and AMR for several years and have published a large number of articles, the same Ethical agreement was used Nr. 2/21 January 2019.

The study was conducted in accordance with the Declaration of Helsinki principles for studies on human beings. The inclusion and exclusion criteria used for sample selection are illustrated in the following:

Inclusion criteria:
Positive urine cultures ≥ 105 CFU/mL;
Identification of a single bacterial pathogen in the culture;
Patients aged 18 years or older.
Exclusion criteria:
Urine cultures < 105 CFU/mL;
Presence of a polymicrobial flora;
Patients with urinary catheters;

Duplicate urine culture results from the same patient during the analyzed period (based on the personal identification number, only the first positive urine culture was registered).

Given that this is a retrospective study and included only outpatient data, medical past, other comorbidities, surgical approach, or baseline pathologies were not included, and the study focused only on comparisons of age, bacterial prevalence, and resistance rates in both sexes.

According to the local policy for both hospitals, treatment in all cases requiring it was administered and guided based on the recommendations of the European Association of Urology (EAU), which are periodically updated [18]. For complex cases requiring close attention, the urologists set the antibiotic treatment in accordance with the infectious disease department’s specifications, following the best available evidence for daily practice.

Urine samples were collected using the standard midstream urine collection technique or by urethral catheterization, depending on the clinical indication. They were transported to the microbiology laboratory within a maximum of two hours of collection, and sample processing followed all standardized microbiological protocols.

Bacterial identification was performed by conventional biochemical methods and, when available, by automated identification systems. Antimicrobial susceptibility testing was performed by the Kirby–Bauer diffusion method on Mueller–Hinton agar, according to the recommendations of the Clinical and Laboratory Standards Institute (CLSI) [19].

The results of the statistical analysis were recorded as R (resistance) and S (susceptibility).

Data were collected and structured using Microsoft Excel (Microsoft Corporation, Redmond, WA, USA; version 16.0). Descriptive statistical analyses were performed in Python version 3.8.9 (Python Software Foundation, Wilmington, DE, USA), using JupyterLab 3.2.4 (Project Jupyter; https://jupyter.org/, 30 March 2026) and the Pandas library 1.3.4 (pandas development team; https://pandas.pydata.org/, 30 March 2026). Categorical variables were expressed as absolute frequencies and percentages. Data comparison between groups regarding antimicrobial resistance rates was assessed with the chi-square test, and when expected cell counts were under 5, Fisher’s test was applied. A *p*-value < 0.05 was considered significant for the compared cases. Grammarly (Grammarly Inc., San Francisco, CA, USA; https://www.grammarly.com/, 30 March 2026)and DeepL (DeepL SE, Cologne, Germany; https://www.deepl.com/translator, 30 March 2026) were used for language accuracy.

## 3. Results

### 3.1. Study Population and Age Distribution

This study included 2.567 patients diagnosed with positive urine cultures, divided into two groups according to their sex. Both inpatient and outpatient urine cultures were analyzed, and the study revealed a higher percentage for the outpatient cases. There were 611 (63.05%) outpatient females vs. 1057 (66.14%) outpatient males, but these data may present less impact because some of the outpatients may also be, or have been hospitalized, while they perform their preoperative and postoperative check-ups in the same center.

The age distribution revealed a clear predominance of old patients in the male group. While in female patients the age distribution was almost equal before 64 years old, in males the UTI incidence dramatically rises over 65 years old, reflecting that 77.47% were diagnosed in the elderly. Overall, the number of male patients over 65 years old was substantially higher (77.47% vs. 46.54%), which may be related to the fact that UTI incidence in men is frequently associated with increased age. These findings are further supported by the differences registered in patients’ mean age (70.5 years in males vs. 59.81 in females).

According to these data, the study reveals a predominance of UTIs in older patients (Table 1).

### 3.2. Uropathogen Distribution

The uropathogen distribution patterns were also analyzed in this study (Table 2). The data obtained suggested a higher prevalence of Gram-negative bacteria in both groups. In females, 80.01% (785) were identified in this category, while in males, there were 76.47% (1222) cases. Gram-positive bacteria were 18.99% (No.= 184) in women and 23.53% (No. = 518) in men, respectively.

There was also a complete bacterial prevalence assessment performed for Gram-negative and positive strains according to age distribution (Table 3). As expected, *E. coli* was predominant in both sexes, but some differences were encountered. Female patients registered a higher prevalence rate of *E. coli* between 18 and 84 years old (over 50%), while in males this was between 31% and 35%. After 85 years the *E. coli* prevalence shifts in females by decreasing to 36.36% which is quite close to that registered in males—31.88%. In contrast, *Klebsiella* spp. were highly registered in men over the 18 to 84 years period, shifting after 85 years with higher prevalence in females (27.27% vs. 14.48%). *Proteus* spp. showed a stable prevalence over the years in both sexes until 85 years, when its prevalence rose to 27.27% in females and 15% in males. For *Pseudomonas* spp. the present data revealed an increasing trend in both sexes over the years starting from very low prevalence in young patients and reaching up to 11–13% after 85 years old.

Among Gram-positive bacteria, *Enterococcus* spp. was identified as the principal agent and showed close results among sexes for young adults, where was registered 11.8% in females and 26.39% in males. This was followed by *Staphylococcus* spp., which also registered similarities regarding bacterial distribution.

### 3.3. Overall Antimicrobial Resistance Rates

#### 3.3.1. Gram-Negative Organisms

Following the results obtained among overall Gram-negative resistance patterns (Table 4), in female patients, the highest results in terms of resistance were obtained for amoxicillin–clavulanic acid (35.77%), levofloxacin (34.71%), and trimethoprim-sulfamethoxazole (34.64%), while fosfomycin (0.38%), meropenem (3.98%), and imipenem (4.44%). The exhibited resistance trends in males also showed increased values, but more pronounced than those found in the other sex. These were illustrated as follows: amoxicillin–clavulanic (50.37%), trimethoprim-sulfamethoxazole (46.69%), and levofloxacin (44.55%). More importantly, male patients registered higher rates of last-resort antibiotics such as imipenem (20.10%) and meropenem (24.02%).

#### 3.3.2. Gram-Positive Organisms

Regarding the identified patterns in Gram-positive patients (Table 5), male patients also showed higher values for several antibiotics compared with the opposite sex. Fluoroquinolones and penicillin showed the highest resistance rates, with higher percentages among males. These were 48.66% for penicillin and 49.16% for fluoroquinolones. One of the positive aspects is that several antibiotic classes, such as linezolid, nitrofurantoin, vancomycin, and fosfomycin, showed a preserved sensitivity profile in both sexes, even though the use of nitrofurantoin and fosfomycin may have limited efficacy in treating male patients.

### 3.4. Species-Specific Antimicrobial Resistance Rates

#### 3.4.1. *Escherichia coli*

*Escherichia coli* was the most frequently identified bacterium in both groups. Regarding antimicrobial resistance patterns (Table 6), higher statistical values were identified in males for several antibiotic classes (Table 6). According to this, *E. coli* showed increased resistance to levofloxacin (45.57% vs. 31.60%), trimethoprim-sulfamethoxazole (45.09% vs. 32.89%), and amoxicillin-clavulanic acid (40.13% vs. 32.62%). Also, statistical differences were illustrated for other antibiotic agents such as aztreonam (17.21% vs. 12.45%) and ceftazidime (17.21% vs. 12.45%).

In contrast to these findings, *E. coli* exhibited preserved antibiotic sensitivity in both sexes to fosfomycin (1.07% vs. 0.40%) and last-line rescue antibiotics like carbapenems, including imipenem (0.83% vs. 0.80%) and meropenem (0.0% vs. 0.21%).

In the present study, these alarming resistance rates may show increased patterns by age, both in outpatient and hospitalized patients, but they tend to offer an overall picture of what’s actually happening in clinical practice.

#### 3.4.2. *Klebsiella* spp.

The results of the resistance and sensitivity comparison in *Klebsiella* spp. are shown in Table 7. According to these data, *Klebsiella* showed even more alarming resistance profiles than *E. coli* in both sexes. The highest resistance in male patients was observed for amoxicillin-clavulanic acid (63.35% vs. 47.22%), followed by trimethoprim-sulfamethoxazole (44.17% vs. 31.08%) and ceftazidime (37.47% vs. 24.50%). Even more worrying results were observed for last resort treatments, such as carbapenems, while the resistance in male patients overpassed 30%.

Also, increased resistance patterns were observed for amikacin (22.11% vs. 10%) and levofloxacin (42.67% vs. 34.9%), but these differences did not reach statistical significance between sexes.

#### 3.4.3. *Pseudomonas* spp.

Unlike the previously characterized bacteria, *Pseudomonas* spp. species analysis did not reveal any statistical differences for most of the compared antibiotic classes, even though increased resistance rates were noticed in both groups (Table 8).

Although statistical analysis did not reach differences, there were registered alarming situations regarding resistance patterns for important antimicrobial agents such as levofloxacin (65.0% in females vs. 49.39% in males), meropenem (47.50% vs. 32.28%), and amikacin (41.03% vs. 28.98%).

Overall, *Pseudomonas* spp. showed high resistance rates in both sexes across multiple antibiotic classes.

#### 3.4.4. *Proteus* spp.

*Proteus* spp. resistance and sensitivity patterns are illustrated in Table 9. Male patients showed statistically significant increases in resistance to amoxicillin-clavulanic acid (53.21% vs. 28.07%) and ceftazidime (22.31% vs. 5.97%) compared with the opposite sex. Also, an important finding was that *Proteus* spp. exhibited increased values in terms of resistance regarding trimethoprim-sulfamethoxazole (60.95% vs. 58.82%) in both sexes.

Amikacin and meropenem registered strong preserved sensitivity in both groups, with resistance rates remaining below 10%.

#### 3.4.5. *Enterococcus* spp.

The most frequently isolated bacteria in Gram-positive, *Enterococcus* spp., showed comparable data in terms of resistance and sensitivity rates between the studied sexes. According to Table 10, high resistance rates were registered for levofloxacin (47.89% in males vs. 48.53% in females) and penicillin (38.64% in males vs. 33.58% in females), while preserved sensitivity was observed for several antibiotics such as fosfomycin (5.53% vs. 7.41%), linezolid (4.10% vs. 6.57%), and nitrofurantoin (6.27% vs. 8.82%). The only statistical difference in resistance was observed with vancomycin, with 2.87% resistance in males and 8.895% in females (*p* = 0.007).

Overall *Enterococcus* spp. species showed large similarities among the studied groups.

#### 3.4.6. *Staphylococcus* spp.

The last bacterial strain discussed in this study, *Staphylococcus* spp., demonstrated significantly higher resistance in males for penicillin (84.93% vs. 54.76%) and levofloxacin (54.05% vs. 24.39%) as it can be seen in Table 11. The most active antimicrobials identified among the studied groups were linezolid and nitrofurantoin.

## 4. Discussion

### 4.1. Epidemiological and Demographic Data

Within this cohort, 77.47% of male patients were aged over 64 years, the male group demonstrating an overall higher age distribution compared to the female population. These data are consistent with the epidemiological profile of urinary tract infections in men, which predominantly manifest at older ages and are often correlated with prostate diseases, urinary obstruction, and more frequent contact with health services [6,8]. This distribution aligns with the clinical profile of a tertiary urological center because male patients, especially those of advanced age, have a higher incidence of complicated urinary tract infections. Several previous studies reported that UTIs in males are frequently associated with urinary lithiasis, benign prostatic hyperplasia, or a history of urological instrumentation; however, given the retrospective nature of this study, including both outpatients and inpatients, these relations were not explored [20,21].

The present study provides a comprehensive evaluation of uropathogen distribution, and one of the main findings is variation in Gram-negative bacterial distribution across the studied groups and time periods.

Regarding the distribution by sex, it remained the most common etiological agent causing UTIs, as well established in the literature; the analysis revealed important differences across age groups in the female population [1]. In contrast to female patients, in whom *E. coli* has a lifetime prevalence exceeding 50%, males exhibited significantly lower rates (31–35%) in the present study. This finding is consistent with previous reports indicating a lower prevalence of this pathogen in male UTIs, particularly those of a complicated nature [6,9]. Over 85 years, a decreasing trend was also observed in females, suggesting that increased age may attenuate sex-related differences. In contrast to *E. coli*, *Klebsiella* analysis revealed a higher prevalence among males, particularly between 18 and 84 years. Literature findings similarly report increased incidence of this pathogen, mostly in complicated and healthcare-associated UTIs [12,22]. An interesting aspect of this study is that *Klebsiella* prevalence shifts over 85 years in females, suggesting that extreme age may represent a risk factor changing the traditional sex-related patterns. In the present study, *Proteus* showed a relatively stable prevalence over the lifetime, with a higher prevalence in the elderly, whereas *Pseudomonas* progressively increased with age. Recent studies have linked this increasing trend in *Pseudomonas* to healthcare-associated UTIs, prolonged hospitalization, and antibiotic exposure [23,24].

Regarding Gram-positive bacteria, *Enterococcus* showed results that were relatively comparable, except in young males, where a slightly increased prevalence was observed. In the literature findings, this appears to be related to complicated and nosocomial UTIs [25].

These findings regarding prevalence underscore that the higher prevalence of non-*E.coli* uropathogens in male patients supports the idea of an individualized approach, based on local epidemiological data and risk factors, as recommended by the European Association of Urology guidelines [18].

### 4.2. Overall Antimicrobial Resistance Comparison

Antibiotic resistance, especially among Gram-negative pathogens, was higher in the male patient group. In particular, resistance to commonly used empirical antibiotics, such as amoxicillin–clavulanic acid, trimethoprim–sulfamethoxazole, and fluoroquinolones, was higher among isolates collected from male patients. This can be highlighted by comparing the amoxicillin–clavulanic acid resistance of the two groups (50.37% in male patients vs. 35.77% in female patients). This aspect is clinically important, given the role of these first-line antibiotics in the treatment of urinary tract infections.

This pattern aligns with data from previous studies showing an increase in resistance to fluoroquinolones and trimethoprim–sulfamethoxazole among uropathogens. In this context, the Infectious Diseases Society of America notes that resistance rates among these bacteria often exceed acceptable limits for empirical therapy [26]. According to Costelloe et al., prior antibiotic use represents a major determinant of antimicrobial resistance at the individual level and may contribute to the increased resistance rates seen in the male cohort [20]. In female patients, susceptibility to aminoglycosides and carbapenems remained relatively preserved. In contrast, male patients exhibited significantly higher rates of carbapenem resistance, with imipenem resistance reaching 20.10%. This difference is clinically important because it indicates a high prevalence of multidrug-resistant Gram-negative bacteria among men. The importance of the results is also supported by the World Health Organization, which classifies carbapenem-resistant Gram-negative bacteria as critical priority pathogens [27].

In the analysis of Gram-positive uropathogens, antibiotic resistance was similar between sexes, with significantly lower variability than that observed in Gram-negative bacteria. However, high resistance rates to fluoroquinolones and penicillin were still noted. Among men, levofloxacin resistance reached 49.16%. In contrast, antibiotics such as fosfomycin, nitrofurantoin, linezolid, and vancomycin maintained high sensitivity and low resistance in both groups analyzed. These findings are consistent with the guidelines of the European Association of Urology, which highlight the stability of antibiotic resistance in Gram-positive bacteria and the need to maintain effective treatment with these agents in the management of urinary tract infections [18].

These findings suggest that Gram-negative uropathogens represent the primary drivers of antimicrobial resistance in urinary tract infections, particularly among male patients, whereas Gram-positive microorganisms exhibit relatively stable susceptibility profiles.

### 4.3. Gram-Negative Antimicrobial Resistance

Recent data support the idea of existing differences related to prevalence and resistance patterns among uropathogens depending on sex. Khanal et al. recently reported an increased rate of multidrug resistance in a large cohort of male patients. This information is also sustained by another study published by McGregor et al. that revealed differences between sexes and also based on age relations, identifying an increased trend in terms of antimicrobial resistance among male patients [28,29]. In the present study, *E. coli* demonstrated increased resistance to several antibiotics, including trimethoprim–sulfamethoxazole, fluoroquinolones, and β-lactams, confirming the literature. For example, *E. coli* exhibited 45.09% resistance to trimethoprim-sulfamethoxazole in men, while 32.89% in females. Together with fluoroquinolones, trimethoprim exceeded the threshold recommended by most of the guidelines for an empirical approach [26]. In previous studies, the significantly higher resistance rates among the studied groups suggest that prior antibiotic exposure may explain the higher resistance rates found in males [20]. In the present study, these resistances may also be influenced by the fact that both outpatients and hospitalized patients accessing dedicated urology services were analyzed.

The actual guidelines suggest that empirical therapy should be adapted to local prevalence patterns, but often highlight that resistance to fluoroquinolones and trimethoprim-sulfamethoxazole is often too high in the general population for these antibiotics to be prescribed [18,26]. The present study revealed alarming situations in the urology setting, especially for male patients. As this study revealed, favorable sensitivity rates, bellow 10%, were found in antibiotics like nitrofurantoin and fosfomycin, which aligns with other reported data regarding these antibiotics’ efficacy against urinary tract infections [30,31]. Unfortunately, these are more specific for treating uncomplicated UTIs, mostly caused by *E. coli*, and exhibit limited activity against other Gram-negative uropathogens. In male patients, urinary tract infections are more often classified as complicated, so their efficacy is very limited.

In the present study, *Klebsiella* spp. revealed high levels of resistance to multiple antibiotic classes, including last resort antibiotics such as carbapenems. In recent reports, carbapenem-resistant *Klebsiella* spp. is presented as a major public threat according to this specific situation, which is severely limiting antibiotic options in clinical management and is also associated with increased morbidity. Compared with lower rates in females, this study found over 30% resistance to imipenem and meropenem in male patients. Recent clinical data in the literature also support the identification of carbapenem-resistant species in complicated urinary tract infections [32].

All this data highlights a higher prevalence of multidrug-resistant strains among male patients, and most recent studies associate this with several factors, such as catheterisation and prior antibiotic administration for obstructive pathologies. They consider antimicrobial exposure and healthcare units as key factors in the development of resistance [21,33].

A special characteristic of this study was the similarities observed between the sexes regarding *Pseudomonas* spp. resistance patterns. These trends revealed increased values for most of the antibiotics tested and, more importantly, the resistance to imipenem exceeded 50% in female cases. Gathering all this data, it may be assumed that *Pseudomonas* spp. resistance may be caused by intrinsic mechanisms or associated with healthcare issues, rather than by sex. Consistent data from the literature suggest specific pathways by which *Pseudomonas* can develop resistance, including chromosomal mechanisms, efflux pumps, antibiotic selection pressure, and biofilm formation [23,24].

The last Gram-negative agent studied, *Proteus* spp., also showed higher resistance rates in male cases. Our data highlighted over 50% resistance to amoxicillin-clavulanic acid in males, whereas significantly lower rates were observed in the opposite sex. Similar reports, like those in the present study, have been reported in the literature regarding increased resistance to β-lactams and trimethoprim–sulfamethoxazole, while preserving sensitivity to carbapenems and aminoglycosides [34,35].

The overall comparison revealed in this study showed higher resistance rates among male patients to several antibiotic classes. *Escherichia coli* and *Klebsiella* registered the most important sex-based differences among the Gram-negative bacteria. The resistance patterns highlight the idea of careful management in male UTIs. In these cases, it is advisable that the empirical treatment strategy be based on the local pattern and adapted to culture-guided management as soon as possible.

### 4.4. Gram-Positive Antimicrobial Resistance

Among Gram-positive bacteria, *Enterococcus* spp. was the main responsible agent for UTIs in both male and female situations, and our data revealed many similarities in antibiotic distribution among several classes. Resistance to fluoroquinolones and penicillin remained high, while high sensitivity was observed for antibiotics such as vancomycin, with a slightly significant increase in females (8.89% vs. 2.87%). Current evidence in the clinical setting also suggests relatively stable patterns in AMR for *Enterococcus* spp. regarding different populations [18]. Also, the high susceptibility rates for nitrofurantoin, fosfomycin, linezolid, and vancomycin are consistent with data from several studies [30,31]. In clinical practice, *Enterococcus* is also recognised as a responsible agent for nosocomial infections. This may explain the present results, given the study design based on outpatient and hospitalized cases.

*Staphylococcus* spp. comparison revealed more significant sex-related differences. Our results confirmed increased penicillin resistance (more than 80%) in males, in contrast to the opposite sex. Also, the resistance to fluoroquinolones was higher in males. This observed pattern is consistent with data from other papers on resistance in *Staphylococcus* related to healthcare management [36].

The data obtained from Gram-positive strains demonstrated a more stable pattern compared to those from Gram-negative strains. Several agents with high efficacy, such as vancomycin and linezolid, can be successfully used in the management of UTIs. This observation is in agreement with the current trends identified by recent studies, which report maintained activity of these agents in Gram-positive bacteria [18,30].

### 4.5. Strengths and Limitations

This study has several strengths, highlighted especially by the large number of cases (2567) of positive urine cultures identified in both male and female subjects. Another important aspect is the bi-center characteristic, comprising two major urology hospitals with different profiles (one for chronic pathology and one for emergency care). The direct comparison of male and female antibiotic resistance is an important strength, as such data are very limited in the specific literature [12]. Analyzing hospitalized and outpatient cases from both sexes underscores the importance of the revealed data by providing a complete picture of day-to-day clinical practice.

On the other hand, several limitations must be acknowledged. First of all, the retrospective design of the study limits the ability to present a complete picture of the clinical data. Several risk factors, such as comorbidities, antibiotic exposure, and influence antimicrobial patterns [5,21]. Another major aspect is that including two tertiary centres may alter the situation by also analyzing complex cases with complicated UTIs. Given that only the first positive urine culture from the same patient was recorded in the present study, a larger analysis including recurrent UTIs or establishing differences between repeated urine cultures could not be performed. Identifying these patients would be a solid base for future evaluation. Also, limited data availability on outpatient cases did not allow a clear distinction between uncomplicated and complicated UTIs. Lastly, including outpatient and hospitalized patients influences the correct differences among the two instances.

## 5. Conclusions

The present findings suggest differences in demographic characteristics and bacterial prevalence between male and female patients.

The overall comparison between sexes illustrated that Gram-negative uropathogens distribution and antimicrobial resistance rates revealed differences, especially *E. coli* and *Klebsiella*, which expressed increased resistance in male patients.

Gram-positive strains exhibited stable resistance patterns in the studied cohort, underscoring the availability of multiple antibiotic options as appropriate treatments in clinical practice.

The present study results may be considered as a basis for developing further analysis regarding sex-age relation with antimicrobial resistance in the Romanian population.

Further data exploring UTI recurrence rates together with multidrug resistance patterns should be considered.

## Figures and Tables

**Table 1:** Age distribution of the study population.

	18–44 Years No. (%.)	45–64 Years No. (%.)	65–84 Years No. (%)	>85 Years No. (%.)	Total
Female	178 (18.37)	340 (35.09)	429 (44.27)	22 (2.27)	969
Male	72 (4.51)	288 (18.02)	1078 (67.46)	160 (10.01)	1598

**Table 2:** Bacterial Gram distribution.

		Female		Male	
Group	Gram	No.	%.	No.	%.
Bacteria	Gram-negative	785	80.01	1222	76.47
	Gram-positive	184	18.99	518	23.53

**Table 3:** Bacterial prevalence according to age distribution.

		18–44		45–64		65–84		>85	
		Female No. (%.)	Male No. (%.)	Female No. (%.)	Male No. (%.)	Female No. (%.)	Male No. (%.)	Female No. (%.)	Male No. (%.)
Gram *−*	*E. coli*	96 (53.93)	23 (31.94)	172 (50.59)	100 (34.72)	248 (57.81)	356 (32.1)	8 (36.36)	51 (31.88)
	*Klebsiella*	30 (16.85)	20 (27.28)	59 (17.35)	70 (24.31)	58 (13.52)	284 (26.35)	6 (27.27)	23 (14.38)
	*Proteus*	18 (10.11)	4 (5.56)	22 (6.47)	17 (5.9)	26 (6.06)	84 (7.79)	1 (27.27)	24 (15.0)
	*Pseudomonas*	1 (0.56)	2 (2.78)	22 (6.47)	36 (12.5)	15 (3.5)	119 (11.04)	3 (13.64)	19 (11.88)
Gram *+*	*Enterococcus*	21 (11.8)	19 (26.39)	51 (15.0)	51 (17.71)	64 (14.92)	198 (18.37)	4 (18.18)	31 (19.38)
	*Staphylococcus*	12 (6.74)	4 (5.56)	14 (4.12)	14 (4.86)	18 (4.2)	47 (4.36)	0(0.0)	12 (7.5)

**Table 4:** Overall, Gram-negative antimicrobial resistance.

Antibiotic	Gram—R		Gram—S		Gram—R		Gram—S	
	Female				Male			
	No.	%.	No.	%.	No.	%.	No.	%.
Amikacin	94	12.27	672	87.73	170	14.3	1019	85.7
Amoxicillin-Clavulanic Acid	259	35.77	465	64.23	473	50.37	466	49.63
Aztreonam	64	19.45	265	80.55	123	31.54	267	68.46
Trimethoprim-Sulfamethoxazole	141	34.64	266	65.36	402	46.69	459	53.31
Ceftazidime	129	16.54	651	83.46	318	27.11	855	72.89
Fosfomycin	2	0.38	528	99.62	14	2.8	486	97.2
Imipenem	33	4.44	710	95.56	121	20.1	481	79.9
Levofloxacin	269	34.71	506	65.29	511	44.55	636	55.45
Meropenem	28	3.98	676	96.02	80	24.02	253	75.98
Nitrofurantoin	55	11.34	430	88.66	167	27.97	430	72.03

**Table 5:** Overall, Gram-positive antimicrobial resistance.

Antibiotic	Gram + R		Gram + S		Gram + R		Gram + S	
	Female				Male			
	No.	%.	No.	%.	No.	%.	No.	%.
Amikacin	6	21.43	22	78.57	1	5.56	17	94.44
Ampicillin	22	15.83	117	84.17	35	13.06	233	86.94
Trimethoprim-Sulfamethoxazole	9	20.93	34	79.07	25	29.76	59	70.24
Fosfomycin	10	7.35	126	92.65	14	5.49	241	94.51
Levofloxacin	76	42.94	101	57.06	176	49.16	182	50.84
Linezolid	11	6.21	166	93.79	13	3.83	326	96.17
Nitrofurantoin	14	8.28	155	91.72	20	5.92	318	94.08
Penicillin	69	38.55	110	61.45	164	48.66	173	51.34
Vancomycin	12	8.7	126	91.3	8	2.87	271	97.13

**Table 6:** *Escherichia coli* antimicrobial resistance and susceptibility rates.

Antibiotic	*E. coli* R		*E. coli* S		*E. coli* R		*E. coli* S		*p* Value
	Female				Male				
	No.	%.	No.	%.	No.	%.	No.	%.	
Amikacin	62	12.11	450	87.89	62	4.82	474	95.18	<0.001
Amoxicillin-Clavulanic Acid	168	32.62	347	67.38	168	40.13	285	59.87	0.016
Aztreonam	23	11.44	178	88.56	23	19.23	105	80.77	0.049
Trimethoprim-Sulfamethoxazole	98	32.89	200	67.11	98	45.09	235	54.91	0.001
Ceftazidime	65	12.45	457	87.55	65	17.21	409	82.79	0.040
Fosfomycin	2	0.4	498	99.6	2	1.07	461	98.93	0.393
Imipenem	4	0.8	497	99.2	4	0.83	238	99.17	1.0
Levofloxacin	164	31.6	355	68.4	164	45.57	264	54.43	<0.001
Meropenem	1	0.21	465	99.79	1	0.0	51	100.0	1.0
Nitrofurantoin	18	4.28	403	95.72	18	9.58	368	90.42	0.004

**Table 7:** *Klebsiella* spp. antimicrobial resistance and susceptibility rates.

Antibiotic	*Klebsiella* R		*Klebsiella* S		*Klebsiella* R		*Klebsiella* S		*p* Value
	Female				Male				
	No.	%.	No.	%.	No.	%.	No.	%.	
Amikacin	15	10.0	135	90.0	86	22.11	303	77.89	0.001
Amoxicillin-Clavulanic Acid	68	47.22	76	52.78	223	63.35	129	36.65	0.001
Aztreonam	24	34.29	46	65.71	45	39.13	70	60.87	0.614
Trimethoprim-Sulfamethoxazole	23	31.08	51	68.92	144	44.17	182	55.83	0.049
Ceftazidime	37	24.5	114	75.5	145	37.47	242	62.53	0.005
Imipenem	4	2.84	137	97.16	60	32.09	127	67.91	<0.001
Levofloxacin	52	34.9	97	65.1	160	42.67	215	57.33	0.124
Meropenem	7	5.0	133	95.0	26	33.33	52	66.67	<0.001
Nitrofurantoin	33	61.11	21	38.89	124	68.51	57	31.49	0.396

**Table 8:** *Pseudomonas* spp. antimicrobial resistance and susceptibility rates.

Antibiotic	*Pseudomonas* R		*Pseudomonas* S		*Pseudomonas* R		*Pseudomonas* S		*p* Value
	Female				Male				
	No.	%.	No.	%.	No.	%.	No.	%.	
Amikacin	16	41.03	23	58.97	51	28.98	125	71.02	0.200
Aztreonam	14	56.0	11	44.0	52	43.33	68	56.67	0.349
Imipenem	20	51.28	19	48.72	52	37.14	88	62.86	0.159
Levofloxacin	26	65.0	14	35.0	81	49.39	83	50.61	0.110
Meropenem	19	47.5	21	52.5	51	32.28	107	67.72	0.106

**Table 9:** *Proteus* spp. antimicrobial resistance and susceptibility rates.

Antibiotic	*Proteus* R		*Proteus* S		*Proteus* R		*Proteus* S		*p* Value
	Female				Male				
	No.	%.	No.	%.	No.	%.	No.	%.	
Amikacin	1	1.54	64	98.46	9	7.14	117	92.86	0.191
Amoxicillin-Clavulanic Acid	16	28.07	41	71.93	58	53.21	51	46.79	0.003
Aztreonam	3	9.09	30	90.91	1	4.0	24	96.0	0.814
Trimethoprim-Sulfamethoxazole	20	58.82	14	41.18	64	60.95	41	39.05	0.984
Ceftazidime	4	5.97	63	94.03	27	22.31	94	77.69	0.007
Imipenem	5	8.06	57	91.94	7	20.0	28	80.0	0.163
Levofloxacin	27	40.3	40	59.7	49	39.84	74	60.16	1.0
Meropenem	1	1.72	57	98.28	3	6.52	43	93.48	0.453

**Table 10:** *Enterococcus* spp. antimicrobial resistance and susceptibility rates.

Antibiotic	*Enterococcus* R		*Enterococcus* S		*Enterococcus* R		*Enterococcus* S		*p* Value
	Female				Male				
	No.	%.	No.	%.	No.	%.	No.	%.	
Ampicillin	21	15.33	116	84.67	35	13.06	233	86.94	0.635
Fosfomycin	10	7.41	125	92.59	14	5.53	239	94.47	0.611
Levofloxacin	66	48.53	70	51.47	136	47.89	148	52.11	0.984
Linezolid	9	6.57	128	93.43	11	4.1	257	95.9	0.400
Nitrofurantoin	12	8.82	124	91.18	17	6.27	254	93.73	0.459
Penicillin	46	33.58	91	66.42	102	38.64	162	61.36	0.375
Vancomycin	12	8.89	123	91.11	8	2.87	271	97.13	0.014

**Table 11:** *Staphylococcus* spp. antimicrobial resistance and susceptibility rates.

Antibiotic	*Staphylococcus* R		*Staphylococcus* S		*Staphylococcus* R		*Staphylococcus* S		*p* Value
	Female				Male				
	No.	%.	No.	%.	No.	%.	No.	%.	
Trimethoprim-Sulfamethoxazole	8	19.05	34	80.95	24	34.29	46	65.71	0.130
Levofloxacin	10	24.39	31	75.61	40	54.05	34	45.95	0.004
Linezolid	2	5.0	38	95.0	2	2.82	69	97.18	0.950
Nitrofurantoin	2	6.06	31	93.94	3	4.48	64	95.52	1.0
Penicillin	23	54.76	19	45.24	62	84.93	11	15.07	<0.001

## Data Availability

Data supporting the reported results are available from the authors.
